# β-Nicotinamide mononucleotide alleviates Alcohol-Induced liver injury in a mouse model through activation of NAD^+^/SIRT1 signaling pathways

**DOI:** 10.1186/s41065-025-00529-x

**Published:** 2025-08-16

**Authors:** Xinxin Yang, Endian Zheng, Yuxian Lin, Haoyue Sun, Ji Zhang, Yingcong Yu

**Affiliations:** 1 Department of Infectious Diseases, Wenzhou Third Clinical Institute Affiliated to Wenzhou Medical University, The Third Affiliated Hospital of Shanghai University, Wenzhou People’s Hospital, Wenzhou, 325000 Zhejiang China; 2 Department of Gastroenterology, Wenzhou Third Clinical Institute Affiliated to Wenzhou Medical University, The Third Affiliated Hospital of Shanghai University, Wenzhou People’s Hospital, Wenzhou, 325000 Zhejiang China; 3 Department of Pharmacy, Wenzhou Third Clinical Institute Affiliated to Wenzhou Medical University, The Third Affiliated Hospital of Shanghai University, Wenzhou People’s Hospital, Wenzhou, 325000 Zhejiang China; 4 School of Medicine, Shanghai University, Shanghai, 200000 Shanghai China

**Keywords:** Β-nicotinamide mononucleotide, Alcoholic hepatic injury, Inflammation, Intestinal permeability, Oxidative stress, Silent information regulator 1

## Abstract

**Background:**

Alcoholism is a significant contributor to the development of alcoholic liver disease, for which no universally accepted and effective treatment currently exists. A precursor of **NAD**^**+**^, β-Nicotinamide mononucleotide (NMN), has revealed potential therapeutic benefits. However, its effectiveness in preventing ethanol-induced liver damage remains uncertain.

**Methods:**

The objective of this study was to assess the protective effects of NMN and elucidate its potential mechanisms using a mouse model subjected to chronic and binge ethanol feeding. Eight-week-old C57BL/6J mice were randomly assigned to one of four groups (*n* = 10 per group): control (CTRL), ethanol (EtOH), ethanol with low-dose NMN (EtOH + NMN(L)), and ethanol with high-dose NMN (EtOH + NMN(H)). Following the completion of the experimental protocol, the mice were euthanized at designated time points, and blood, liver, and ileum tissues were collected for analysis of relevant biomarkers.

**Results:**

Compared to the CTRL group, the EtOH group demonstrated increased liver specific gravity and elevated blood ALT levels. Administration of NMN improved histopathological changes in the liver and ileum of the mice. NMN significantly counteracted the ethanol-induced elevation in liver MDA levels and restored the diminished glutathione (GSH) and superoxide dismutase (SOD) activity levels caused by ethanol exposure. Additionally, NMN inhibited the ethanol-induced expression of cytochrome P450 2E1 (CYP2E1). It also reduced the release of pro-inflammatory cytokines, including TNF-α, IL-6, and IL-1β, which were triggered by ethanol exposure, improved energy homeostasis in the ileum, and reversed the downregulation of mRNA and protein expression of key tight junction proteins in the ileum, specifically ZO-1, Claudin-1, and Occludin, thereby restoring their functional integrity. Furthermore, NMN activated the **NAD**^**+**^/ **SIRT1** signaling pathway, leading to the upregulation of all target genes.

**Conclusion:**

NMN supplementation provides protection against alcoholic liver injury in a mouse model, potentially through the upregulation of the cellular **NAD**^**+**^/ **SIRT1** pathway. This upregulation enhances antioxidant and anti-inflammatory activities and improves intestinal permeability.

**Supplementary Information:**

The online version contains supplementary material available at 10.1186/s41065-025-00529-x.

## Introduction

Alcoholism represents a significant global public health challenge, posing serious risks to human health [1]. Ethanol is primarily metabolized in the liver, while the gut, as the main site for ethanol absorption, is also highly susceptible to damage from excessive alcohol consumption. Alcoholic liver disease (ALD) encompasses a spectrum of liver damage and impaired liver function resulting from prolonged heavy drinking, including conditions such as fatty liver, steatohepatitis, liver fibrosis, cirrhosis, and hepatocellular carcinoma [2]. ALD is among the leading causes of end-stage liver disease, and its progression is closely associated with oxidative stress, inflammation, and mitochondrial dysfunction, including imbalances in the **NAD**^**+**^/NADH ratio [3, 4]. A decline in hepatic **NAD**^**+**^ levels due to ethanol exposure is recognized as a key contributor to alcoholic liver injury [5]. 

The intestinal barrier is composed of mechanical, chemical, immunological, and microbial components, serving as a critical defense to prevent the transfer of intestinal contents and metabolites into the circulation. Ethanol induces liver damage by changing gut microbiota, impairing the function of the intestinal barrier, and allowing the translocation of intestinal contents and metabolites into the bloodstream [6]. Thus, identifying substances that can maintain intestinal barrier integrity is key for the prevention and treatment of ethanol-induced damage.

While alcohol abstinence remains the most effective measure, current pharmacological therapies may provide some benefits to patients with ALD; however, these treatments are frequently associated with adverse side effects. Nicotinamide mononucleotide (NMN), a precursor of **NAD**^**+**^, is a biologically active nucleotide that exists in two isomeric forms, α and β, with the β form being the active variant [7]. NMN contributes to various physiological processes through its conversion to **NAD**^**+**^, including the activation of SIRT1, **NAD**^**+**^-dependent class III protein deacetylase, which regulates cellular survival and apoptosis. NMN has demonstrated pharmacological potential in the treatment of multiple diseases, with NMN supplementation revealed to increase hepatic **NAD**^**+**^ levels [8, 9]. In early-stage models of ALD, NMN supplementation effectively reduces ethanol-induced elevations in alanine aminotransferase (ALT) and aspartate aminotransferase (AST) levels [10]. Moreover, ethanol exposure leads to a reduction in **NAD**^**+**^ levels in the ileum, while supplementation with **NAD**^**+**^ precursors increases **NAD**^**+**^ levels in the ileum and alleviates ethanol-induced damage to the intestinal epithelial barrier, indicating a protective role for **NAD**^**+**^ precursors in gut health [11]. Hence, NMN holds promise as a potential therapeutic agent for ALD. However, few foundational studies have focused on the protective effects of NMN on intestinal barrier function in mouse models of alcoholic liver injury. To examine the impact of NMN on ethanol-induced liver damage, this study used the NIAAA model, a well-established mouse model of chronic and binge ethanol feeding.

## Materials and methods

### Animals

Eight-week-old male C57BL/6J mice were obtained from SPF (Beijing) Biotechnology Co., Ltd, under Certificate no. SCXK (Beijing) 2019-0010. The mice were housed in a specific pathogen-free (SPF) facility with a 12-hour light/dark cycle (Certificate no. SYXK (Zhe) 2021-0005). The room temperature was maintained at 23 ± 5 °C, and the relative humidity at 60 ± 15%. All animal procedures were assessed and approved by the Animal Ethics Committee of Wenzhou Medical University (Certificate no. wydw2023-0373).

The mice were randomly assigned to one of four groups (*n* = 10): control group, ethanol group, ethanol plus low-dose NMN supplementation group (300 mg/(kg·d)), and ethanol plus high-dose NMN supplementation group (500 mg/(kg·d)). To acclimate the mice to the liquid diet, a control liquid diet was provided for five days. Subsequently, all groups except the CTRL group were fed a 5% ethanol liquid diet for 10 days. On day 16, ethanol-fed mice received a single dose of ethanol (5 g/kg body weight) by gavage in the early morning, while the CTRL group was fed the control liquid diet and gavaged with isocaloric maltose dextrin. NMN was administered daily via intraperitoneal injection at doses of 300 mg/kg or 500 mg/kg, the selection of these dosage levels, administration route, and frequency was based on comprehensive considerations including preliminary research data, safety evaluations, and bioavailability factors [10, 12, 13]. Studies have demonstrated that NMN administration (300 mg/kg/day for 1 year) did not induce any significant toxic or adverse effects. Excessively high doses may cause adverse reactions or even death in mice, while excessively low doses may fail to produce observable experimental effects. To assess safety in a preliminary manner, we monitored body weight, general behavior, and liver histology in the CTRL + NMN(H) group (*n* = 5), and observed no signs of toxicity (data not shown). The chosen doses of 300 mg/kg and 500 mg/kg not only ensure detectable experimental outcomes but also maintain safety and feasibility throughout the study. Nine hours after gavage, the mice were anesthetized with isoflurane gas, and blood samples were collected. While under general anesthesia, the mice were euthanized, and the liver was collected and weighed. The left lobe of the liver and a section of the ileum were fixed in 4% paraformaldehyde, while the remaining liver and ileum tissues were snap-frozen in liquid nitrogen and stored at -80 °C.

### Materials

β-Nicotinamide mononucleotide NMN was obtained from Yuyao Laifusiben Health Technology Co., Ltd. The Liber-DeCarli liquid diet was provided by Bio-Serv. Maltodextrin was sourced from MACKUN, and anhydrous ethanol was acquired from Anhui Ante Food Co., Ltd. Serum ALT and AST levels were measured using a commercial kit (ALT Reagent Kit (Mindray, Cat# 105-000442-00);AST Reagent Kit (Mindray, Cat# 105-000443-00); TC (Total Cholesterol) Reagent Kit (Mindray, Cat# 105-000448-00);TG (Triglycerides) Reagent Kit (Mindray, Cat# 105-000449-00)) on a Beckman Coulter AU480 automated chemistry analyzer. ALT、AST: values are expressed in U/L, which corresponds to international units of enzyme activity per liter of serum (1 U = 1 µmol substrate converted per minute at 37 °C). Detection kits for adenosine triphosphate (ATP), adenosine diphosphate (ADP), tumor necrosis factor-α (TNF-α), interleukin-6 (IL-6), interleukin-1β (IL-1β), malondialdehyde (MDA), superoxide dismutase (SOD), glutathione (GSH), and coenzyme I (nadide) (H) were purchased from Beijing Suolaibao Biotechnology Co., Ltd. Antibodies for cytochrome P-450 (CYP2E1), silent information regulator 1 (SIRT1), nicotinamide phosphoribosyltransferase (NAMPT), and zonula occludens-1 protein (ZO-1) were obtained from Boster Bioengineering Co., Ltd. Additionally, antibodies for Claudin-1, Occludin, and nicotinamide mononucleotide adenylyl transferase (NMNAT) were purchased from Proteintech, USA.

### Drug Preparation and administration

A β-nicotinamide mononucleotide solution was prepared by mixing 300 mg or 500 mg β-nicotinamide mononucleotide powder with 10 ml of water. Then, 6.6 ml of 95% ethanol was mixed with 13.4 ml of water to prepare a 31.5% (vol/vol) ethanol solution. Lastly, 9 g of maltodextrin was dissolved in a final volume of 20 ml of water to prepare a 45% (wt/vol) maltodextrin solution.

### General condition of mice and the liver specific gravity

Throughout the experiment, behavioral changes in the mice were observed, and their body weights were monitored daily. The livers of the mice were weighed, and the liver-specific gravity was calculated.

### Histological analysis

Liver and ileum tissues were immersed in 4% paraformaldehyde, followed by embedding in paraffin and sectioning at a thickness of 3 μm. The sections underwent dewaxing and hydration processes, after which they were stained using hematoxylin and eosin (H&E). Pathological abnormalities in the liver and ileum were examined under high magnification (×200).

### Biochemical assays

ALT and AST serum levels were measured using an automated biochemical analyzer(Mindray, CL-6000i). Blood **NAD**^**+**^ and NADH levels were assessed with an **NAD**^**+**^/NADH assay kit. Hepatic triglycerides (TG) and cholesterol (TC) were also detected using an automated biochemical analyzer, while hepatic levels of MDA, GSH, SOD, TNF-α, IL-6, and IL-1β were measured using ELISA kits.

### Western blot analysis

RIPA extraction buffer was used for tissue lysis, followed by precise determination of protein concentration using a BCA protein assay kit. Protein samples were separated using SDS-PAGE, and the target protein band was transferred on to a PVDF membrane. The membrane was then blocked for 30 min. Incubation with primary antibodies specific to the target protein was conducted overnight at 4 °C. Subsequently, secondary antibodies were applied to the membranes for two hours at room temperature. After a further wash with TTBS, the membrane was treated with ECL solution for visualization. Protein levels of CYP2E1, SIRT1, NAMPT, and NMNAT in liver tissue, as well as ZO-1, Claudin-1, and Occludin in ileum tissue, were assessed using glyceraldehyde-3-phosphate dehydrogenase (GAPDH) as an internal control. ImageJ software (National Institute of Health, Bethesda, MD, USA) was used for the analysis.

### RT-qPCR

Following the collection of ileum tissues, total RNA was extracted using the Trizol method. Reverse transcription was conducted according to the instructions provided with the reverse transcription kit, followed by amplification of the target genes using quantitative PCR (q-PCR). The 2-ΔΔCt method was used to quantify the mRNA levels of ZO-1, Claudin-1, and Occludin in the ileum, with GAPDH used as the internal reference gene. The primer sequences are depicted in Table 1.

**Table 1:** Quantitative real-time PCR primers

Gene		Primer sequence (5’-3’)
ZO-1	F	AAGATGGGATTCTTCGGCCC
	R	CTTGGCTGCAGGGCTATCTT
Claudin-1	F	GCCTTGATGGTAATTGGCATCC
	R	GGCCACTAATGTCGCCAGAC
Occludin	F	TAAGTCAACACCTCTGGTGCC
	R	TTCCTGCTTTCCCCTTCGTG
GAPDH	F	TCAGCCGCATCTTCTTTTGC
	R	CCCAATACGACCAAATCCGT

### Measurement of metabolites

ATP and ADP levels in ileum tissues were measured using an Agilent HPLC 1260 series (Agilent, USA). ATP Standard Solution Preparation: ATP powder (1 vial): Store at -20 °C.Before use: Add 1.8 mL distilled water to prepare a 1 µmol/mL ATP stock solution, then store at -20 °C.Dilution series: Dilute the 1 µmol/mL ATP stock solution with distilled water to prepare 0.5, 0.1, 0.05, 0.01, and 0.005 µmol/mL ATP standard solutions.ADP Standard Solution Preparation: ADP powder (1 vial): Store at -20 °C.Before use: Add 2.34 mL distilled water to prepare a 1 µmol/mL ADP stock solution, then store at -20 °C.Dilution series: Dilute the 1 µmol/mL ADP stock solution with distilled water to prepare 0.5, 0.1, 0.05, 0.01, and 0.005 µmol/mL ADP standard solutions.Tissue Homogenization: Weigh 0.3 g tissue sample, add 1.5 mL Extraction Buffer I, and homogenize in an ice bath. Incubate in an ice bath for 40 min. First Centrifugation: Centrifuge at 10,000 rpm, 4 °C for 10 min. Collect 750 µL supernatant. Second Extraction: Add 750 µL Extraction Buffer II, vortex vigorously for 5 min. Centrifuge again at 10,000 rpm, 4 °C for 10 min. Filtration: Filter the supernatant through a water-based syringe filter into an amber HPLC vial. Keep at room temperature and analyze within 2 h. HPLC Analysis Setup Instrument Preparation: Turn on the computer and HPLC system. Install the HPLC column and launch the chromatography software. Method Parameters: Injection volume: 10 µL Column temperature: 27 °C Flow rate: 0.8 mL/min Detection wavelength: 254 nm Run time: 70 min Mobile Phase: Composition: Acetonitrile : Mobile Phase B (pH 6.15) = 2 : 98 Column equilibration: Flush the column until the baseline stabilizes. Standard and Sample Analysis Standard Solution Injection: Inject 10 µL of each ATP/ADP standard solution. Retention times: ADP: ~6.7 min ATP: ~7.8 min Sample Injection: Inject 10 µL of the prepared sample solution. Record peak areas for ATP and ADP at their respective retention times. Quantification: Calculate ATP and ADP concentrations based on standard curves. Key Notes Stability: ATP/ADP standards should be prepared fresh or stored at -20 °C (avoid repeated freeze-thaw cycles). HPLC Maintenance: After analysis, flush the column with high-purity water to prevent salt precipitation. Data Validation: Ensure linearity (R² ≥ 0.99) for standard curves. This protocol ensures accurate and reproducible quantification of ATP and ADP in biological samples using HPLC.

### Statistical analysis

Data were presented as mean ± standard deviation (SD). Normal distribution data were analyzed using one-way analysis of variance (ANOVA), while non-normal distribution data were assessed using the Kruskal-Wallis test. Statistical analyses and visualizations were conducted using GraphPad Prism version 9.0 software and ImageJ (https://imagej.nih.gov/ij/). A significance level of *p* < 0.05 was established for determining statistically significant differences.

## Results

### NMN effectively significantly improved the ethanol-induced liver histopathological changes

**Fig. 1:**
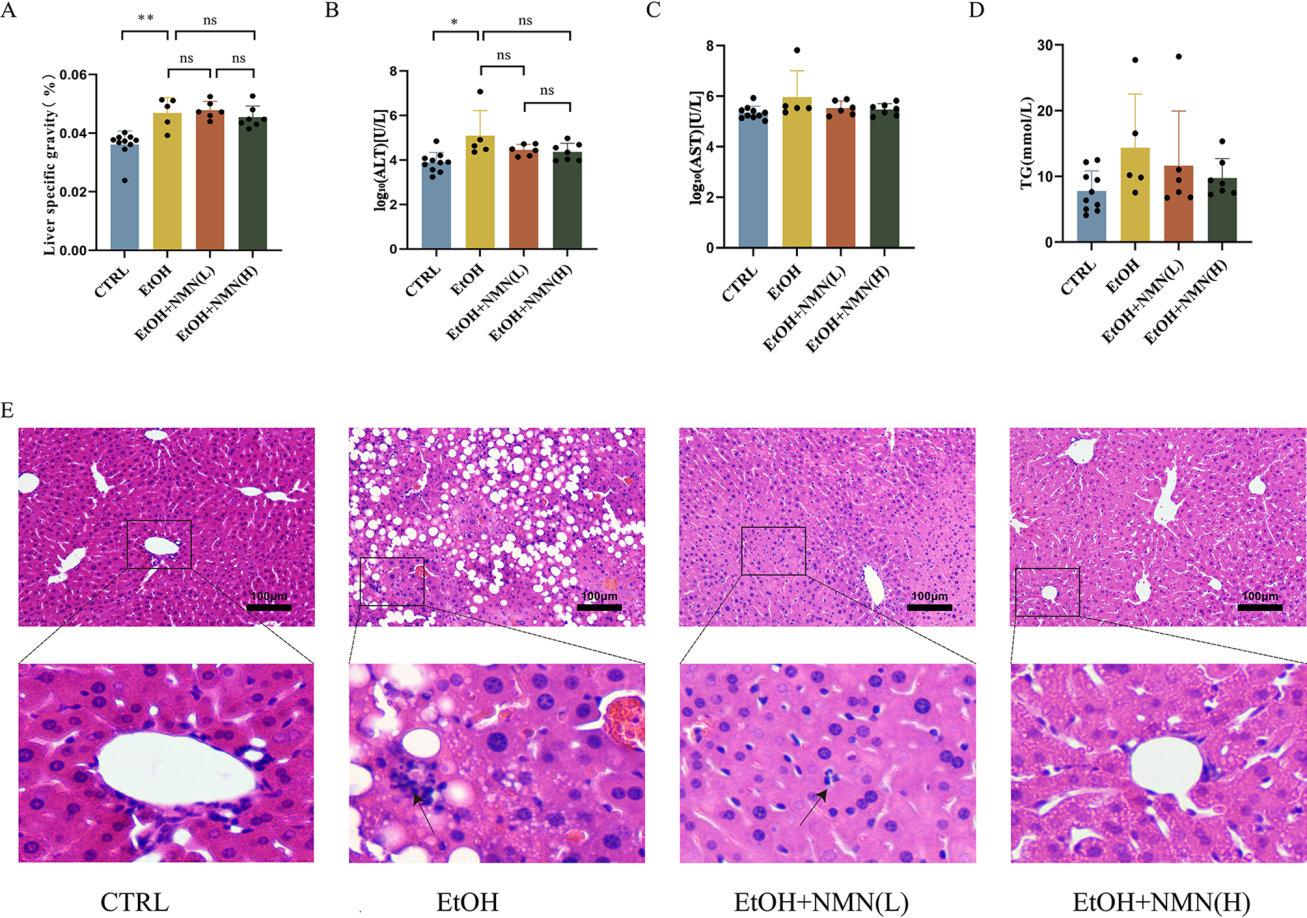
(**A**) Liver Specific Gravity. (**B**) Serum ALT levels. (**C**) Serum AST levels. (**D**) Liver TG level. (**E**) Images of mouse liver with H&E staining at 200x magnification. Data is presented as mean ± SD (*n* = 5–10 per group). *p < 0.05, **p < 0.01, ***p < 0.001

Compared to the control group, an increase in liver specific gravity was observed with ethanol exposure; however, NMN supplementation did not result in a reduction. Following logarithmic transformation, serum ALT levels were elevated due to ethanol exposure, while NMN supplementation did not produce a significant reduction. No significant differences in serum AST levels were noted among the four groups. Ethanol exposure led to an increase in liver TG, while NMN supplementation resulted in a reduction; however, this difference was not statistically significant (*P* > 0.05). Histological analysis via H&E staining of liver sections revealed that the basic architecture of liver cells in the ethanol group was disrupted, with evident steatosis, enlargement of liver cells, and infiltration of inflammatory cells, as indicated by black arrows. In contrast, NMN supplementation significantly mitigated the ethanol-induced histopathological changes in the liver, demonstrating relatively normal hepatocyte morphology, orderly arranged hepatic cords, improved fatty degeneration, and a marked decrease in inflammatory cell infiltration, as indicated by the black arrows (Fig. 1).

### NMN reduces oxidative stress and inflammatory response in mice with ALD

**Fig. 2:**
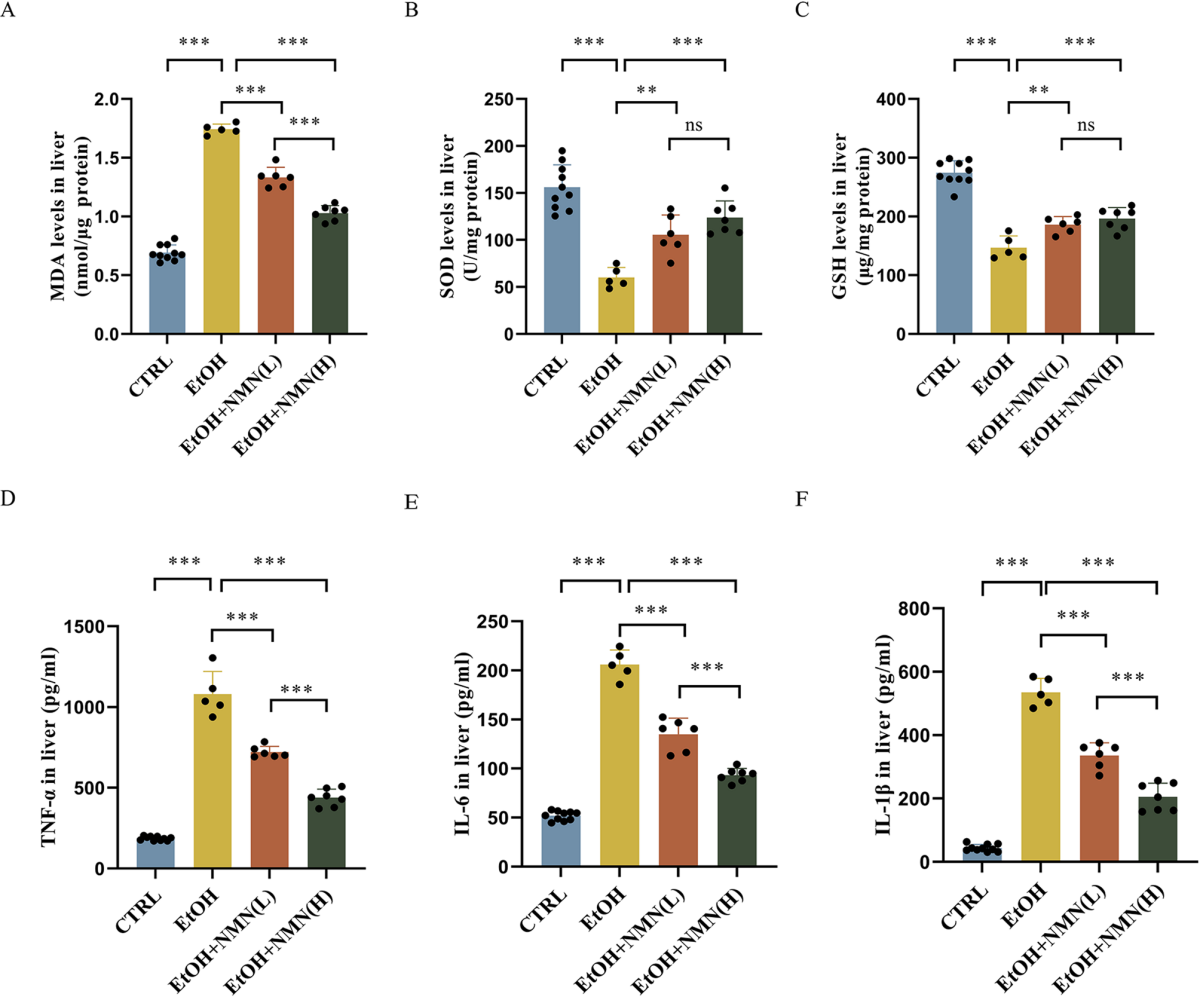
(**A**) Liver MDA levels. (**B**) Liver SOD levels. (**C**) Liver GSH levels. (**D**) Liver TNF-α(level). (**E**) Liver IL-6 levels. (**F**) Liver IL-1β levels

In the pathogenesis of alcoholic liver disease (ALD), oxidative stress and inflammation play critical roles. Compared with the control group, ethanol-fed mice exhibited significantly increased hepatic malondialdehyde (MDA) levels and significantly decreased superoxide dismutase (SOD) activity and glutathione (GSH) levels (all *P* < 0.001). In comparison with the ethanol group, NMN supplementation significantly reduced hepatic MDA levels (*P* < 0.001), with the high-dose NMN group showing a more pronounced effect than the low-dose NMN group (*P* < 0.001). Additionally, NMN treatment increased SOD activity and GSH content compared to the ethanol group (low-dose NMN group: *P* < 0.01; high-dose NMN group: *P* < 0.001). These results indicate that NMN supplementation significantly suppresses hepatic oxidative stress. Compared with the control group, ethanol exposure upregulated the expression of pro-inflammatory cytokines, including TNF-α, IL-6, and IL-1β (all *P* < 0.001). In contrast, NMN-treated mice showed significantly lower levels of TNF-α, IL-6, and IL-1β compared to the ethanol group (all *P* < 0.001). Moreover, the high-dose NMN group exhibited a more significant reduction than the low-dose NMN group (*P* < 0.001), suggesting that NMN alleviates alcohol-induced hepatic inflammation in mice, with the high-dose NMN group providing superior protective effects. These findings demonstrate that NMN effectively mitigates alcohol-induced liver injury by suppressing oxidative stress and inflammation, with higher doses yielding more pronounced benefits. (Fig. 2).

### NMN prevents ethanol induced metabolic disorders

**Fig. 3:**
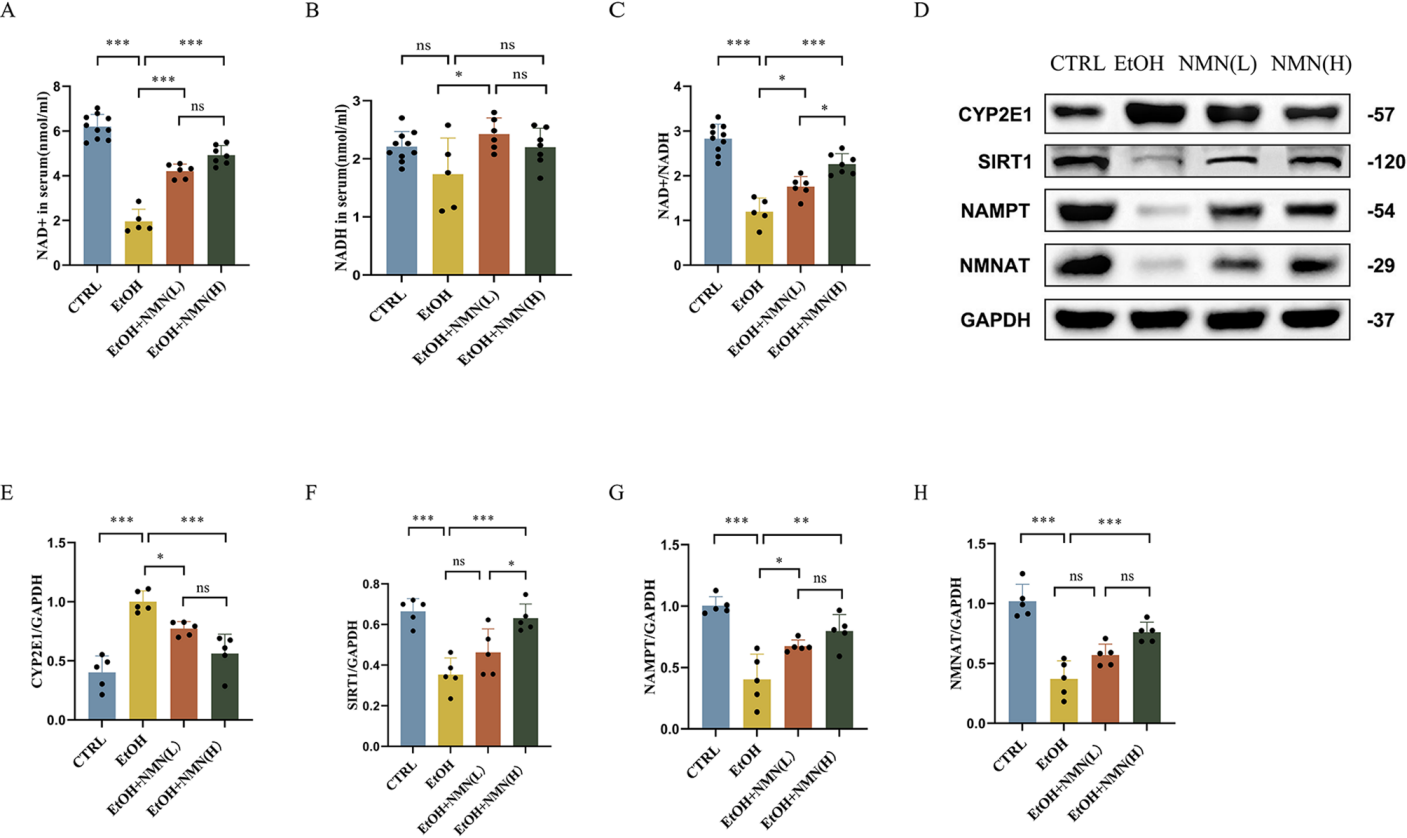
(**A**) Serum **NAD**^**+**^ levels. (**B**) Serum NADH levels. **C**) The **NAD**^**+**^/NADH ratio. (**D**-**H**) Western blots of CYP2E1, SIRT1, NAMPT, and NMNAT, with GAPDH as an internal control (*n* = 5). (**E**-**H**) Image J was used to quantify the proteins CYP2E1, SIRT1, NAMPT, and NMNAT

Compared with the control group, ethanol exposure significantly decreased serum NAD + levels (*P* < 0.001), while NMN supplementation reversed this ethanol-induced decline (all *P* < 0.001). Additionally, ethanol reduced the serum NAD+/NADH ratio compared to the control group (*P* < 0.001), and NMN administration restored this ratio (low-dose NMN group: *P* < 0.05; high-dose NMN group: *P* < 0.001). The high-dose NMN group exhibited a more significant increase in the NAD+/NADH ratio than the low-dose group (*P* < 0.05). In terms of protein expression, ethanol upregulated hepatic CYP2E1 protein levels compared to the control group (*P* < 0.001), whereas NMN treatment downregulated CYP2E1 expression (low-dose NMN group: *P* < 0.05; high-dose NMN group: *P* < 0.001). Furthermore, ethanol decreased SIRT1 protein expression relative to the control group, and high-dose NMN significantly increased SIRT1 levels (all *P* < 0.001). Ethanol also reduced hepatic NAMPT protein expression compared to the control group (*P* < 0.001). NMN supplementation upregulated NAMPT expression (low-dose NMN group: *P* < 0.05; high-dose NMN group: *P* < 0.01), though there was no significant difference between the two NMN doses (*P* > 0.05). Similarly, ethanol decreased NMNAT protein expression, while high-dose NMN significantly elevated NMNAT levels (all *P* < 0.001) (Fig. 3).

### NMN prevents ethanol induced ileal pathological changes and energy imbalance in mice

**Fig. 4:**
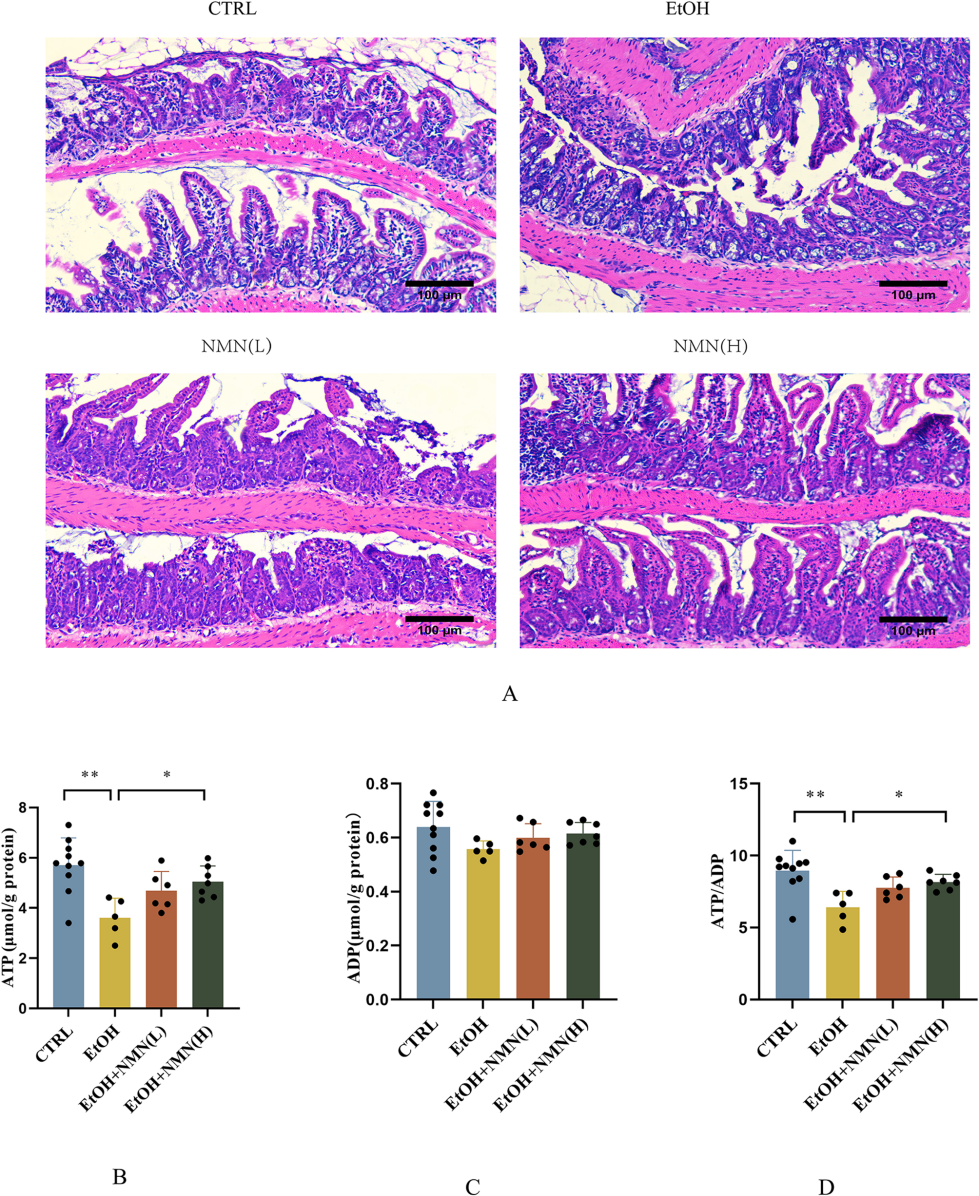
: (**A**) Representative histological image of an ileum sample, including H&E images at 200× magnification. (**B**) Ileal ATP level. (**C**) Ileum ADP levels. (**D**) The ratio of ATP/ADP

H&E staining of the ileum sections revealed that ethanol exposure disrupted the intestinal epithelial cell structure and resulted in an incomplete villi structure, as indicated by the black arrows. Additionally, the crypt structure was absent, with inflammatory cell infiltration and fibrous tissue hyperplasia, as highlighted by the red arrows. In contrast, the NMN supplement group exhibited intact villi and epithelial cell structures. These findings indicate that NMN supplementation significantly mitigated the ethanol-induced histopathological changes in the ileum. Ethanol exposure also significantly decreased ileal ATP levels and the ATP/ADP ratio, whereas high-dose NMN supplementation effectively restored energy balance, as demonstrated by increased ATP levels and an improved ATP/ADP ratio (Fig. 4).

### NMN attenuates ethanol-induced intestinal barrier injury

**Fig. 5:**
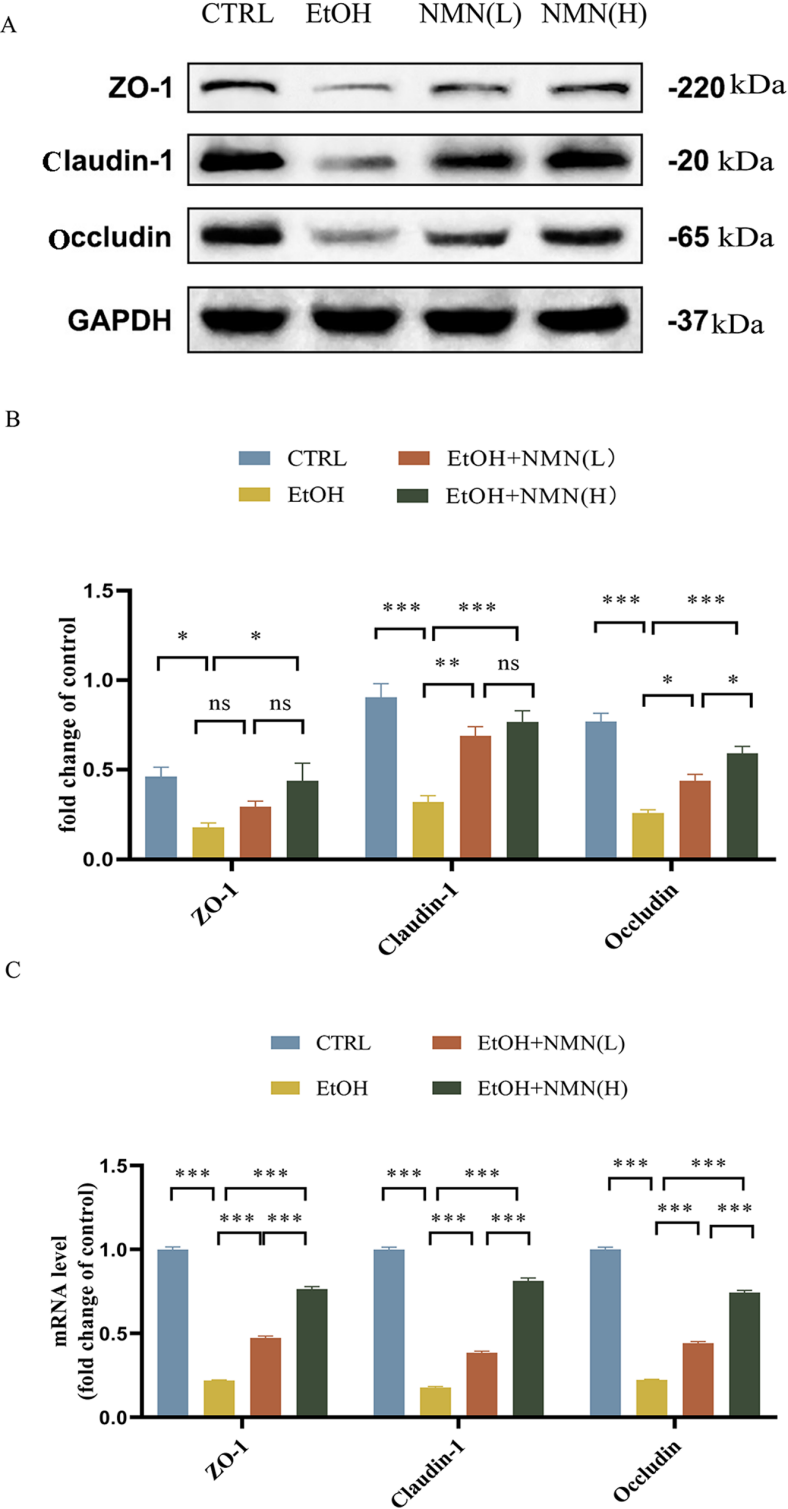
: (**A**) Western blots of ZO-1, Claudin-1, and Occludin, with GAPDH as an internal control (*n* = 5). (**B**) Protein quantification of ZO-1, Claudin-1, and Occludin using Image J.(**C**) The relative mRNA levels of the ZO-1, Claudin-1, and Occludin genes in the mouse ileum (*n* = 5–10)

Compared with the control group, ethanol exposure significantly downregulated the relative protein expression of ZO-1 in the ileum, while high-dose NMN supplementation upregulated ZO-1 expression (both *P* < 0.05). Ethanol treatment downregulated ileal Claudin-1 protein expression compared to controls (*P* < 0.001), whereas NMN administration restored Claudin-1 levels (low-dose NMN group: *P* < 0.01; high-dose NMN group: *P* < 0.001). However, no statistically significant difference was observed between the low- and high-dose NMN groups (*P* > 0.05). Similarly, ethanol significantly reduced Occludin protein expression in the ileum versus controls (*P* < 0.001), while NMN treatment upregulated Occludin expression (low-dose group: *P* < 0.05; high-dose group: *P* < 0.001). Notably, the high-dose NMN group demonstrated significantly stronger therapeutic effects compared to the low-dose group. The mRNA levels of ZO-1, Claudin-1, and Occludin were reduced in the EtOH group compared to the CTRL group, NMN supplementation effectively reversed the ethanol-induced decrease in the expression of these proteins. The high-dose NMN group demonstrated significantly stronger effects compared to the low-dose NMN group. (Fig. 5).

## Discussion

ALD is a liver disease caused by prolonged excessive alcohol consumption, for which no approved pharmacological treatment currently exists. NMN has emerged as a promising candidate for ALD therapy. This study used the National Institute on Alcohol Abuse and Alcoholism (NIAAA) model to replicate the drinking behaviors of patients with ALD, characterized by chronic alcohol consumption combined with a single binge-drinking episode [14]. To assess the effects of NMN supplementation in a mouse model of early-stage ALD, various techniques were used, including WB, PCR, and H&E staining. The results indicate that NMN provides a protective effect against alcohol-induced liver injury in mice, primarily through the activation of SIRT1. As a precursor of **NAD**^**+**^, NMN helps maintain intracellular **NAD**^**+**^ balance, enhances SIRT1 activity, mitigates oxidative stress, increases anti-inflammatory responses, and reduces intestinal barrier damage, thereby playing a protective role in this ALD mouse model.

**NAD**^**+**^ serves as a vital coenzyme in mitochondrial oxidative phosphorylation and energy metabolism, playing a role in numerous biological processes such as metabolism, aging, apoptosis, DNA repair, and gene expression [15, 16]. In the liver, ethanol is primarily metabolized by alcohol dehydrogenase into acetaldehyde, which is subsequently oxidized by acetaldehyde dehydrogenase to acetate. This metabolic process depletes **NAD**^**+**^ and results in an excess production of NADH, leading to a reduced **NAD**^**+**^/NADH ratio in hepatocytes [17]. This imbalance contributes significantly to ethanol-induced steatosis, oxidative stress, steatohepatitis, and insulin resistance [10]. Thus, strategies that restore the **NAD**^**+**^/NADH ratio by enhancing **NAD**^**+**^ synthesis could offer therapeutic potential for ethanol-induced liver damage. NAMPT is the rate-limiting enzyme in the **NAD**^**+**^ salvage pathway and is essential for maintaining intracellular **NAD**^**+**^ levels. NAMPT catalyzes the conversion of nicotinamide to NMN, which is subsequently transformed into **NAD**^**+**^ by NMNAT. As demonstrated by research, NAMPT influences the activity of SIRT1, which is dependent on **NAD**^**+**^. Ethanol has been revealed to inhibit NAMPT expression in the liver, while NAMPT overexpression can significantly elevate intracellular **NAD**^**+**^ levels, thereby alleviating ethanol-induced hepatic steatosis in mice [18]. 

**NAD**^**+**^ biosynthesis involves several enzymes, including NMNAT, which plays a role in synthesizing mitochondrial NAD. Overexpression of NMNAT can elevate **NAD**^**+**^ levels and activate Sirt3, leading to improved mitochondrial biogenesis, energy metabolism, and restoration of mitochondrial function [19]. Studies have highlighted the importance of **NAD**^**+**^ deficiency, resulting from suppressed NMNAT expression, in doxorubicin-induced hepatotoxicity models in mice. Supplementation with NMN mitigates liver injury and oxidative stress [20]. 

Therefore, activation of NAMPT and NMNAT may represent a promising strategy to attenuate alcohol-related liver injury. In this study, NMN supplementation effectively restored the protein expression levels of NAMPT and NMNAT, which were diminished due to ethanol exposure, and reversed the ethanol-induced decline in **NAD**^**+**^ levels and the **NAD**^**+**^/NADH ratio.

Among the seven human SIRT subtypes, SIRT1 is the most extensively studied. As a **NAD**^**+**^-dependent enzyme, SIRT1 activation is regulated by intracellular **NAD**^**+**^ levels. Ethanol-induced reduction in SIRT1 expression can influence the activity of various transcription factors and co-regulators, including AMPK, SREBP-1, PGC-1α, and Lipin-1. These changes can downregulate several metabolic and inflammatory pathways in the liver, such as fatty acid β-oxidation, de novo fatty acid synthesis, lipoprotein uptake and secretion, and the production of pro-inflammatory cytokines [21, 22]. Extensive evidence indicates that SIRT1 plays a key role in regulating lipid metabolism and inflammatory responses in both rodent and human livers, and that the impairment of SIRT1 signaling is closely associated with the progression of ALD [21]. 

In experimental models of ALD, SIRT1 expression at the protein or mRNA level is consistently suppressed, indicating that modulating SIRT1 activity may represent a crucial mechanism for the prevention of ALD [23]. In the current study, NMN supplementation effectively restored the protein expression of SIRT1. The increase in serum **NAD**^**+**^ levels observed with NMN supplementation is likely attributed to the reversal of ethanol-induced suppression of NAMPT and NMNAT expression—two key enzymes in the **NAD**^**+**^ biosynthesis salvage pathway. This upregulation of SIRT1 protein expression contributed to the protective effects of NMN against alcoholic liver injury.

Alcohol-induced hepatotoxicity is predominantly mediated by acetaldehyde [24]. Acetaldehyde contributes to oxidative stress by reducing GSH levels and promoting the formation of ROS [25, 26]. Oxidative stress plays a key role in ethanol-induced liver injury, as it can enhance fatty acid synthesis, disrupt mitochondrial function, induce cellular stress, and ultimately lead to cell death [27–30]. Thus, mitigating ethanol-induced oxidative stress is critical for preventing ALD. Normally, there is a balance between prooxidants and antioxidants. Chronic alcohol consumption disrupts this equilibrium, leading to excessive production of free radicals and a significant increase in MDA levels. This imbalance results in severe oxidative stress, hepatocyte damage, and apoptosis. MDA serves as an indicator of lipid peroxidation, thereby indirectly reflecting the extent of free radical-induced damage to liver tissue [31]. In contrast, GSH and SOD possess robust antioxidant properties [32]. Alcohol consumption impairs both enzymatic and non-enzymatic antioxidant defenses, rendering liver cells more vulnerable to ROS-induced damage. The findings of this study indicated that ethanol exposure not only elevated MDA levels but also reduced GSH levels and SOD activity in a model of alcoholic liver injury. The data indicates that NMN supplementation may reduce ethanol-induced damage by enhancing the antioxidant system and counteracting oxidative stress.

ADH in the liver is primarily responsible for converting ethanol into acetaldehyde, which is subsequently oxidized to acetate by ALDH. An additional pathway involved in ethanol metabolism is the microsomal ethanol oxidation system (MEOS), which relies on cytochrome P450 enzymes, particularly CYP2E1 [2]. According to research, chronic alcohol consumption elevates the expression of CYP2E1, leading to an overproduction of ROS. These ROS can interact with DNA and proteins, causing damage and exacerbating oxidative stress, ultimately resulting in hepatocyte injury [33]. CYP2E1 is recognized as a key contributor to ethanol-induced liver damage, and reducing the activity of CYP2E1 or the oxidative stress it generates can mitigate the hepatotoxic effects of ethanol [34]. CYP2E1 inhibitors such as disulfiram have demonstrated protective effects against ethanol-induced liver injury [35]. In this study, the expression of CYP2E1 in liver tissue was assessed to further elucidate the mechanism of NMN in mitigating liver injury. The results indicated that NMN treatment significantly suppressed the ethanol-induced upregulation of CYP2E1 in mice. These findings indicate that NMN may decrease ROS production and enhance antioxidant defense by inhibiting CYP2E1 expression, thereby providing protection against alcohol-induced liver damage.

In addition to oxidative stress, a prominent characteristic of ALD is the inflammatory response. Chronic alcohol consumption activates Kupffer cells, which subsequently increases the release of proinflammatory cytokines, including TNF-α and IL-6 [36]. The excessive release of these cytokines contributes to immune dysregulation and cytokine imbalance, ultimately resulting in liver injury [37]. In the present study, ethanol exposure led to elevated levels of TNF-α, IL-6, and IL-1β, whereas NMN supplementation significantly reduced the secretion of these proinflammatory cytokines. These findings indicate that NMN may confer protection against alcohol-induced liver injury by mitigating inflammatory processes.

Moreover, it has been proposed that alcohol consumption enhances the synthesis and release of pro-inflammatory factors like TNF-α, IL-1β, and IL-6, by increasing intestinal permeability and promoting bacterial translocation. This process allows endotoxins, such as lipopolysaccharides (LPS), to enter the liver via the portal vein [38]. Alcohol also heightens the sensitivity of Kupffer cells and hepatocytes to LPS and TNF-α, resulting in a detrimental enterohepatic inflammatory cycle [39]. Cellular energy homeostasis plays a key role in the formation and maintenance of tight junctions (TJs) within the intestinal epithelium, thereby preserving the integrity of the intestinal barrier [40, 41]. While limited research has been conducted on the relationship between NMN and intestinal health, some studies have indicated that NMN can reduce intestinal mucosal permeability and protect intestinal integrity [42]. 

In the current study, NMN supplementation effectively prevented disruptions in ATP levels and the ATP/ADP ratio in the ileum, thereby alleviating the energy imbalance induced by ethanol. The ethanol group exhibited a significant reduction in the mRNA levels and protein expression of ZO-1, Claudin-1, and Occludin compared to the control group. However, NMN supplementation mitigated this decline, suggesting that NMN may protect against alcoholic liver injury by preserving energy balance and maintaining intestinal barrier function.

Serum ALT and AST levels serve as crucial and sensitive biomarkers for assessing liver function; their abnormal elevations indicate hepatocyte injury and necrosis. However, in this study, NMN did not reverse the ethanol-induced increase in ALT levels, likely due to the short duration of the modeling, which may not have provided sufficient time for NMN to exert a more pronounced protective effect. The early-stage model of ethanol toxicity used in this study may not capture all liver pathological changes. Thus, using a more severe model of alcoholic liver disease could yield additional insights.

Although NMN did not significantly reduce serum ALT levels in this early-stage ALD model, marked histological improvement was observed. This discrepancy may reflect the temporal dissociation between enzyme leakage (ALT) and structural recovery. ALT elevation often peaks within hours of acute injury, whereas histological repair may lag. Additionally, ALT is a sensitive but not always specific marker of hepatocellular damage, especially in mild or early injury. Thus, the histopathological findings, combined with improvements in oxidative stress and inflammation, suggest that NMN exerts hepatoprotective effects that may not yet be fully reflected in serum ALT levels. In addition, ALT levels can be measured at multiple time points to monitor potential improvement.While ALT is a sensitive marker for hepatocyte injury, it primarily reflects disruptions in cell membrane integrity. If NMN preferentially mitigates oxidative stress or inflammation, significant changes in enzymatic indicators may not be evident.In summary, the protective effects of NMN may predominantly involve chronic, subcellular-level improvements (e.g., mitochondrial function, anti-inflammatory effects) rather than acute membrane damage.

While we demonstrated that NMN improves ileal tight junction integrity and energy balance, direct measures of gut-derived endotoxin (e.g., plasma LPS), bacterial translocation markers (e.g., 16 S rDNA in mesenteric lymph nodes), or portal cytokine levels were not included. These endpoints are currently being evaluated in a follow-up study using a chronic-plus-binge ethanol model to more directly link intestinal barrier restoration with hepatic inflammation.

In summary, the findings of this study indicate that NMN protects against alcoholic liver injury in a mouse model, primarily through mechanisms involving reduced oxidative stress, suppression of the inflammatory response, and enhancement of intestinal permeability, all of which are mediated by modulation of the **NAD**^**+**^/SIRT1 signaling pathways. As a critical mediator of the gut-liver axis, NMN may influence the onset and progression of alcoholic liver disease, offering new perspectives for treatment strategies. Therefore, NMN presents as a promising health product warranting further investigation. Additional research is necessary to elucidate the effects of NMN on the pathophysiology of alcoholic liver disease and to determine optimal dosage, frequency, and administration methods.

## Supplementary Information

Below is the link to the electronic supplementary material.

Supplementary Material 1

## Data Availability

All data generated or analysed during this study are included in this article. Further enquiries can be directed to the corresponding author.

## Abbreviations

NMN: (nicotinamide mononucleotide)
ALD: (Alcoholic liver disease)
NAD^+^: (Nicotinamide adenine dinucleotide)
SIRT1: (Sirtuin 1)
NMNAT: (Nicotinamide mononucleotide adenylyl transferase)
NAMPT: (Nicotinamide Phosphoribosyltransferase)
CYP2E1: (Cytochrome P-450 2E1)
ALT: (Alanine aminotransferase)
AST: (Aspartate aminotransferase)
TC: (Cholesterol)
TG: (Triglyceride)
MDA: (Malondialdehyde)
SOD: (Superoxide dismutase)
GSH: (Glutathione)
PCR: (Polymerase Chain Reaction)
WB: (Western Blotting )
ELISA: (Enzyme-Linked Immunosorbent Assay)
HE: (Hematoxylin-eosin)
TNF-α: (Tumor necrosis factor-α)
IL-6: (Interleukin-6)
IL-1β: (Interleukin-1β)
ROS: (Reactive oxygen species)
GAPDH: (Glyceraldehyde-3-phosphate dehydrogenase)
ATP: (Adenosine Triphosphate)
ADP: (Adenosine Diphosphate)
ZO-1: (Zonula Occludens-1 Protein)
PMSF: (Phenylmethylsulfonyl fluoride)
RIPA: (Radio immunoprecipitation assay)
SDS: (Sodium dodecyl sulfate)
TEMED: (Tetramethylethylenediamine)
TBST: (Tris-Buffered Saline with Tween-20)
PVDF: (Polyvinylidene fluoride)
TBS: (Tris Buffered Saline)
SDS-PAGE: (Sodium dodecyl sulfate - polyacrylamide gelelectrophoresis)
