# Supplementary figures and images for: β-Nicotinamide mononucleotide alleviates Alcohol-Induced liver injury in a mouse model through activation of NAD+/SIRT1 signaling pathways
Source: Hereditas. 2025 Aug 16;162:161. doi: 10.1186/s41065-025-00529-x (PMC12357478; doi:10.1186/s41065-025-00529-x)

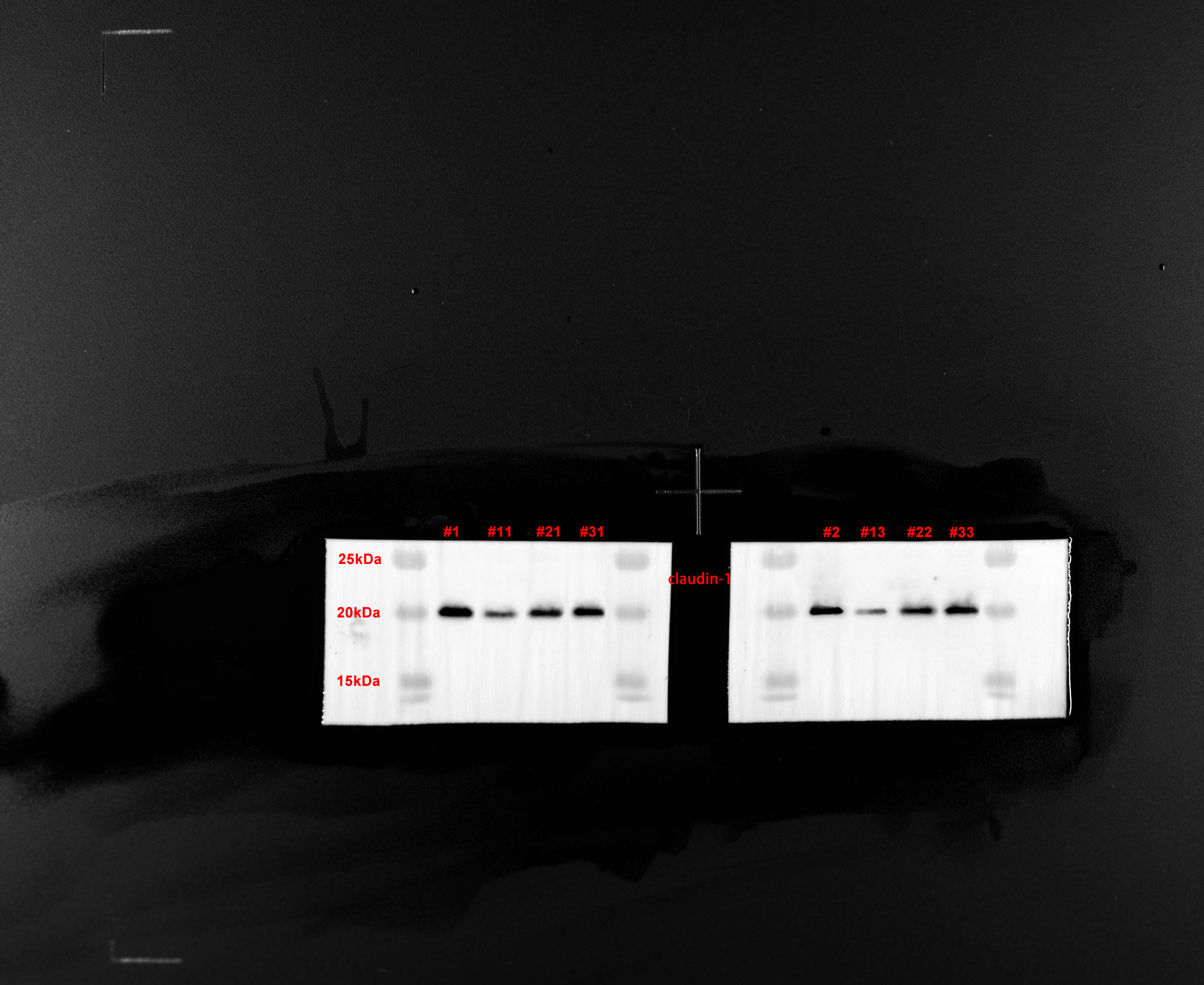

Supplement: Supplementary file 1 — Supplementary Material 1 [file 41065_2025_529_MOESM1_ESM.zip › raw data/claudin-1(1,11,21,31,2,13,22,33)+marker.jpg]

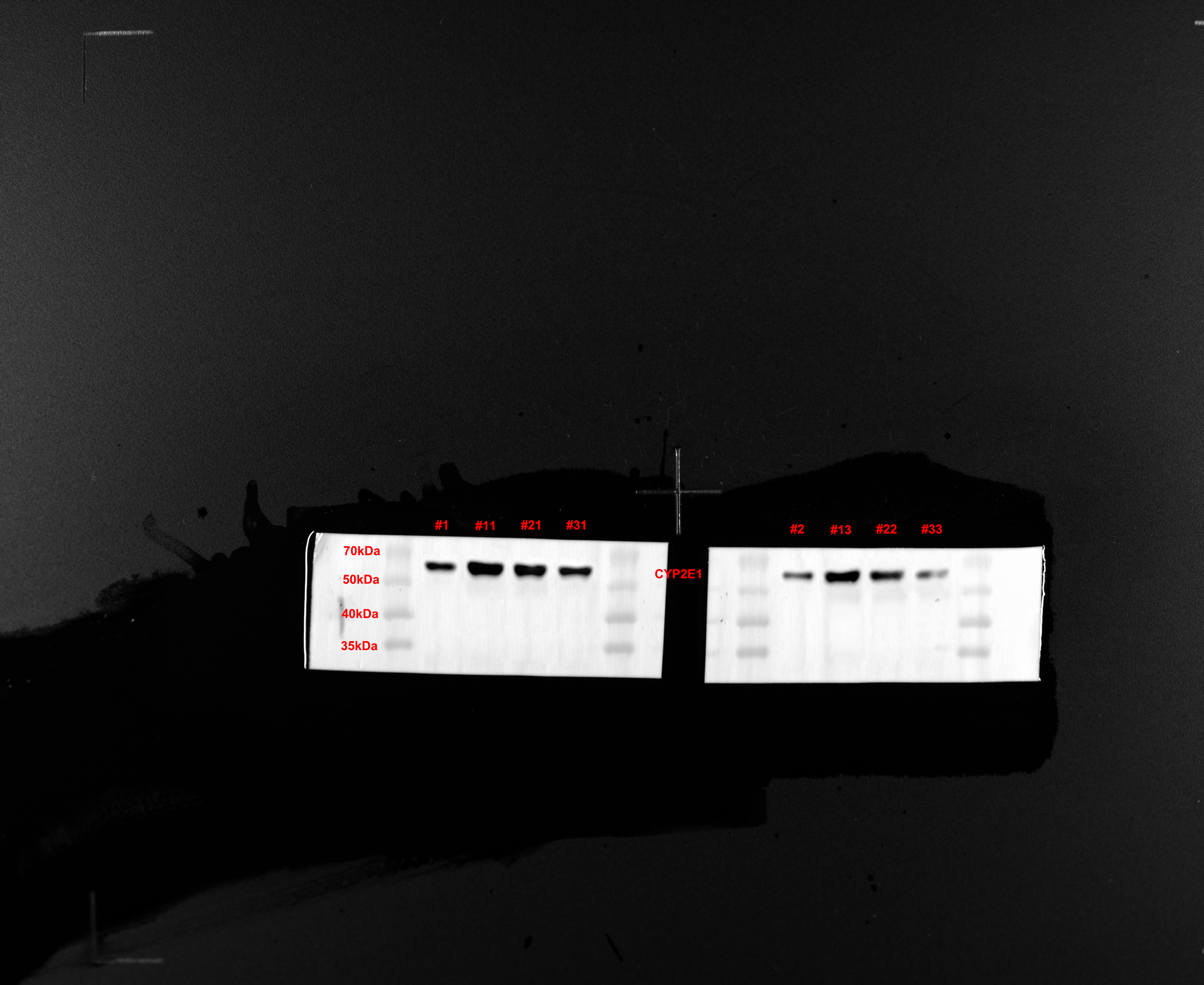

Supplement: Supplementary file 1 — Supplementary Material 1 [file 41065_2025_529_MOESM1_ESM.zip › raw data/CYP2E1(1,11,21,31,2,13,22,33)+marker.jpg]

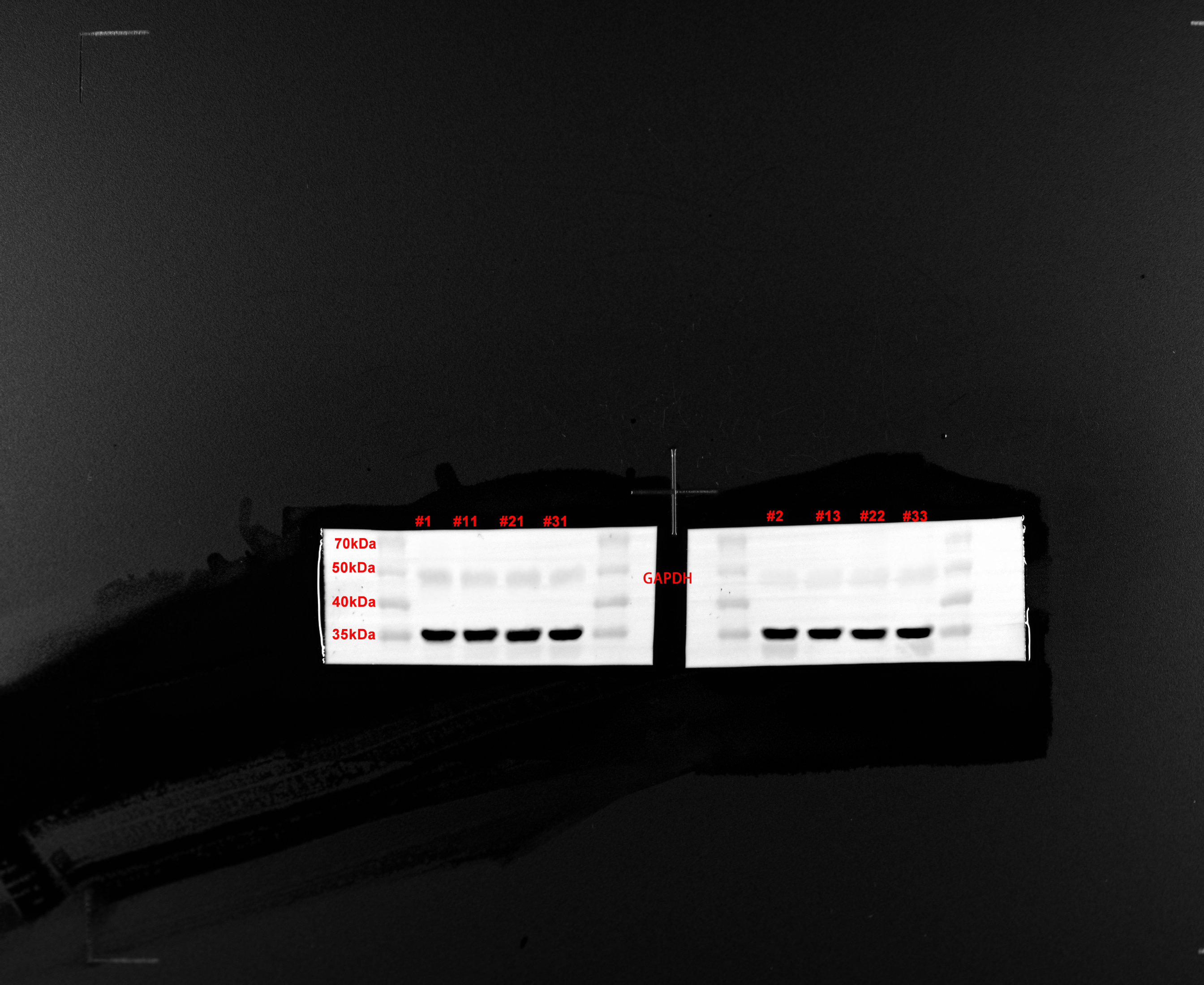

Supplement: Supplementary file 1 — Supplementary Material 1 [file 41065_2025_529_MOESM1_ESM.zip › raw data/GAPDH(1,11,21,31,2,13,22,33)A+marker.jpg]

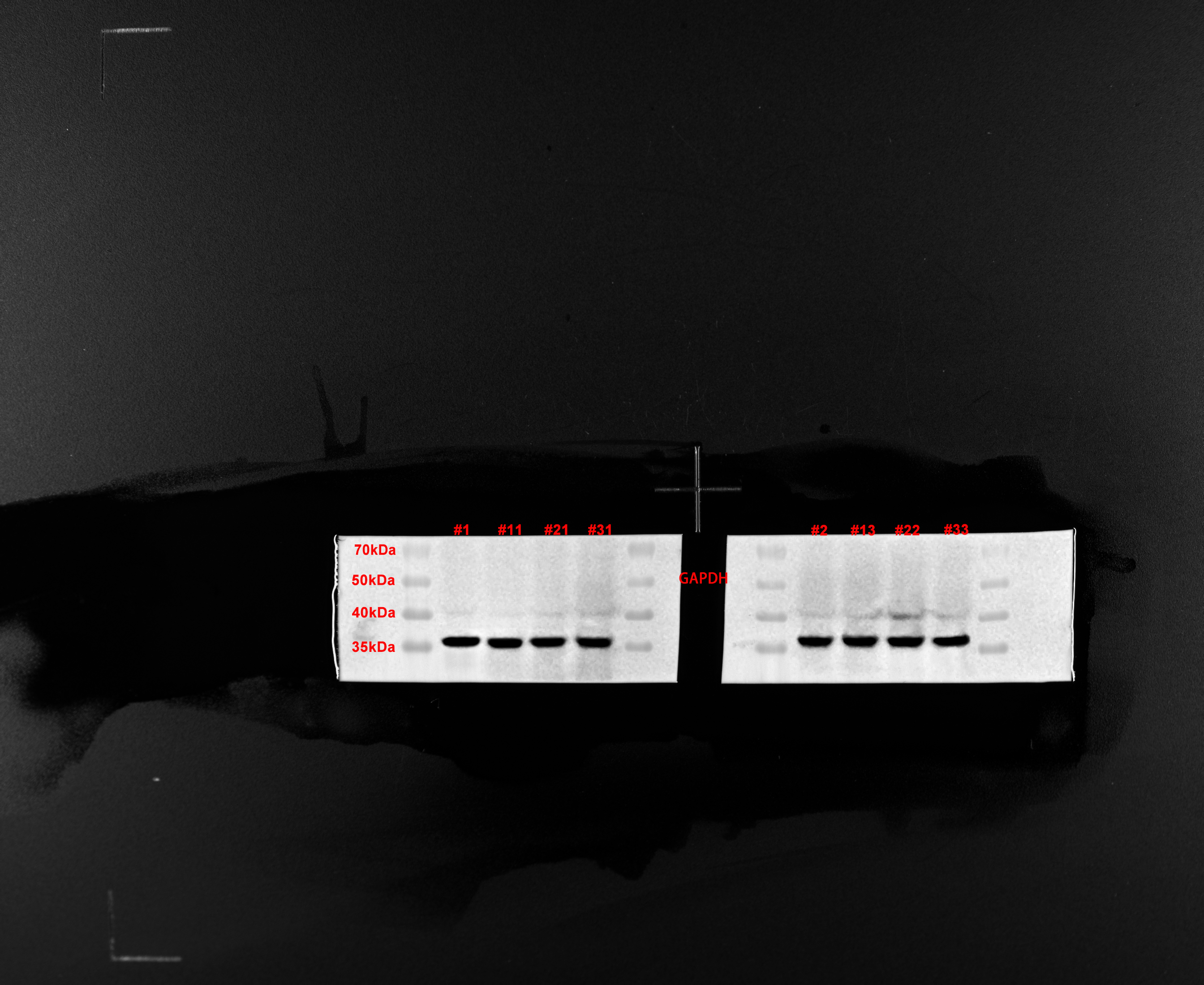

Supplement: Supplementary file 1 — Supplementary Material 1 [file 41065_2025_529_MOESM1_ESM.zip › raw data/GAPDH(1,11,21,31,2,13,22,33)+marker.jpg]

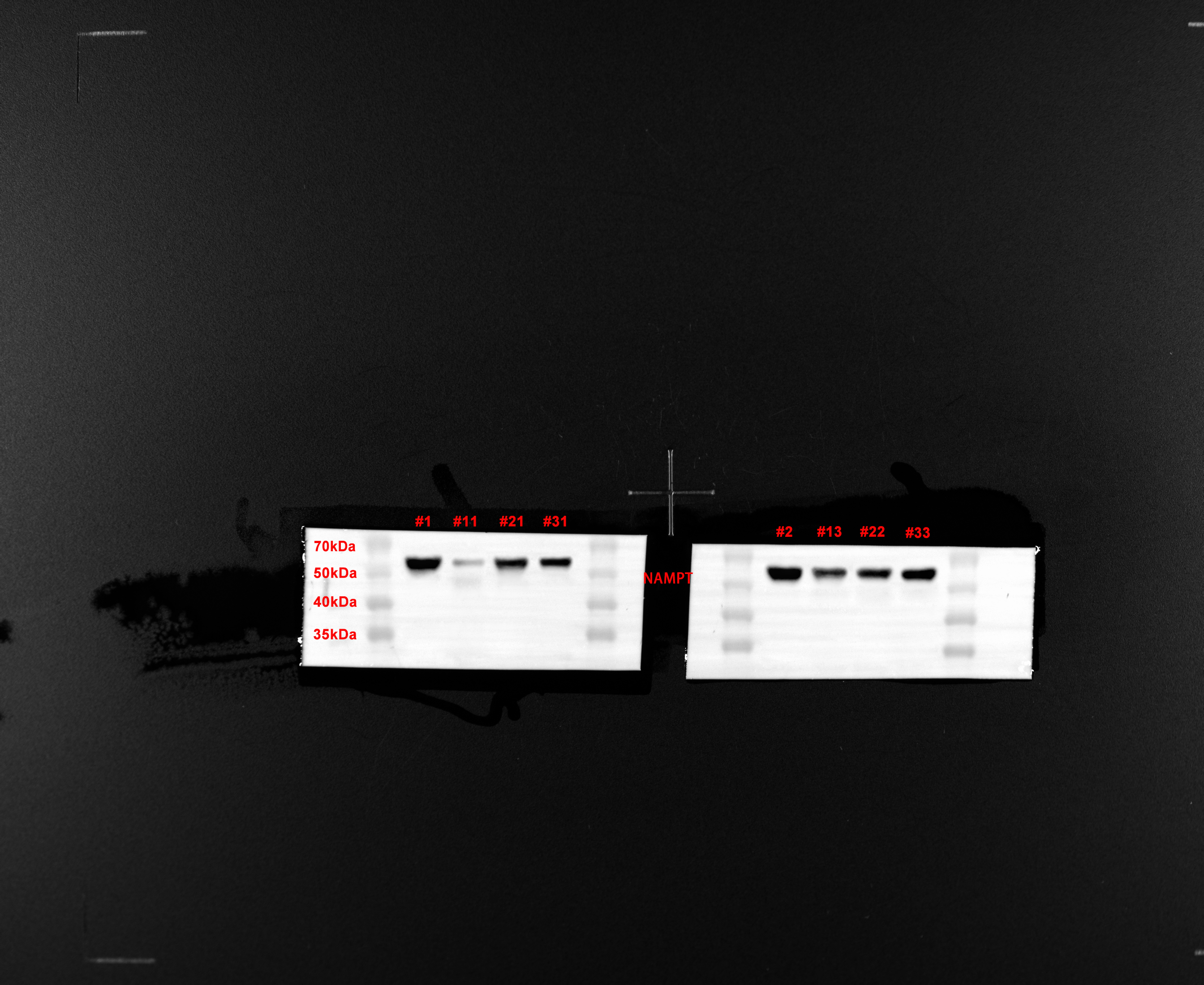

Supplement: Supplementary file 1 — Supplementary Material 1 [file 41065_2025_529_MOESM1_ESM.zip › raw data/NAMPT(1,11,21,31,2,13,22,33)+marker.jpg]

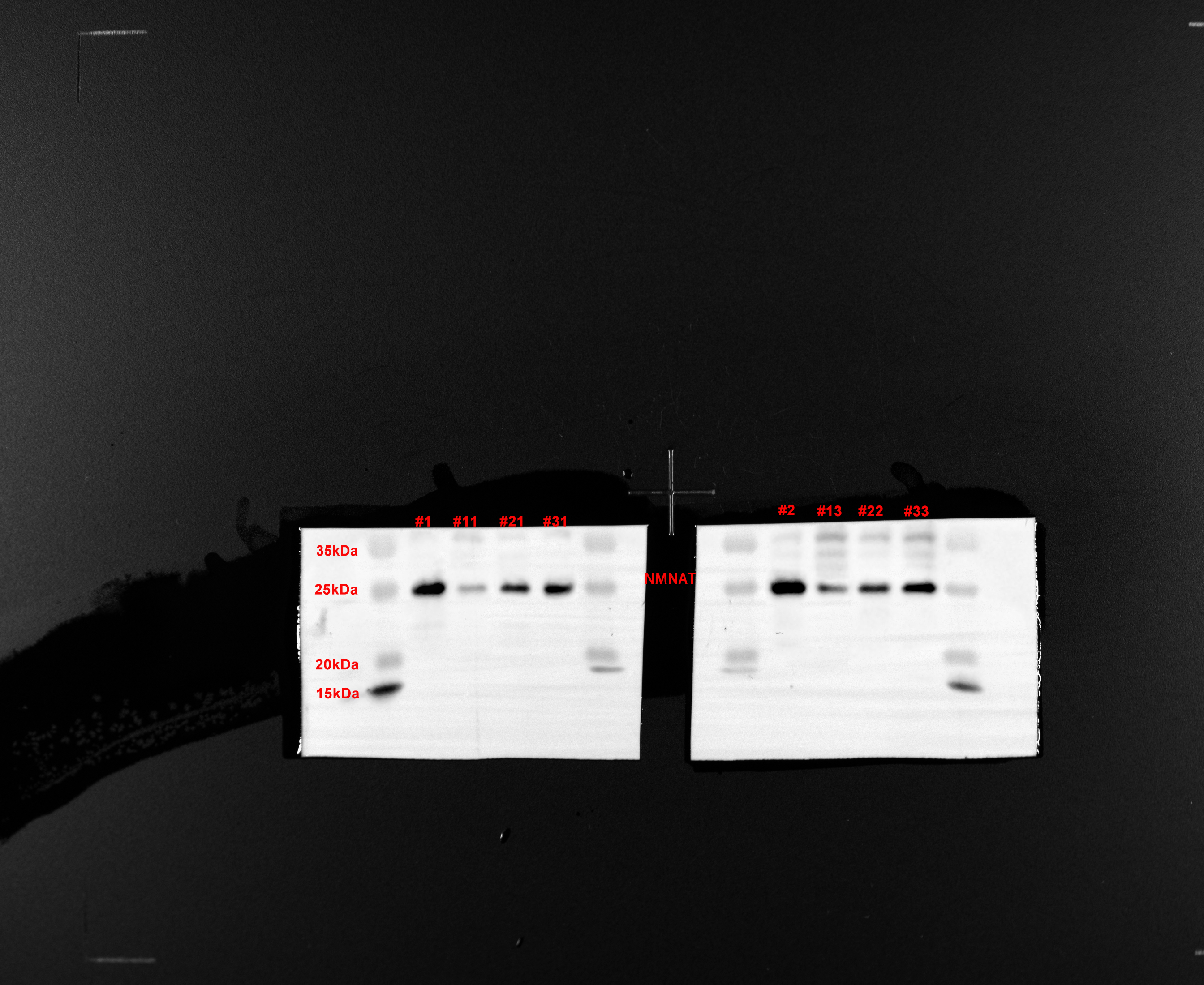

Supplement: Supplementary file 1 — Supplementary Material 1 [file 41065_2025_529_MOESM1_ESM.zip › raw data/NMNAT(1,11,21,31,2,13,22,33)+marker.jpg]

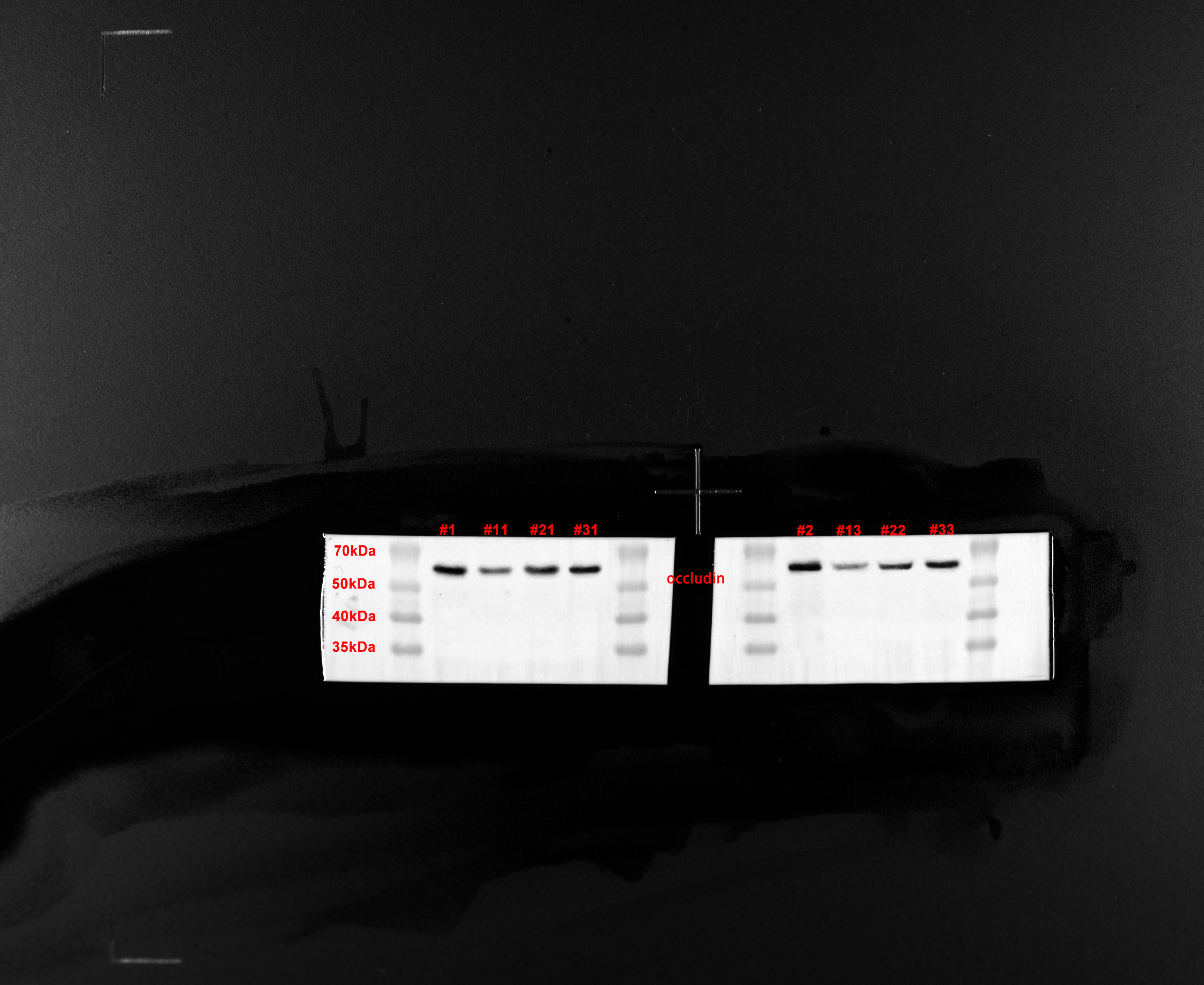

Supplement: Supplementary file 1 — Supplementary Material 1 [file 41065_2025_529_MOESM1_ESM.zip › raw data/occludin(1,11,21,31,2,13,22,33)marker.jpg]

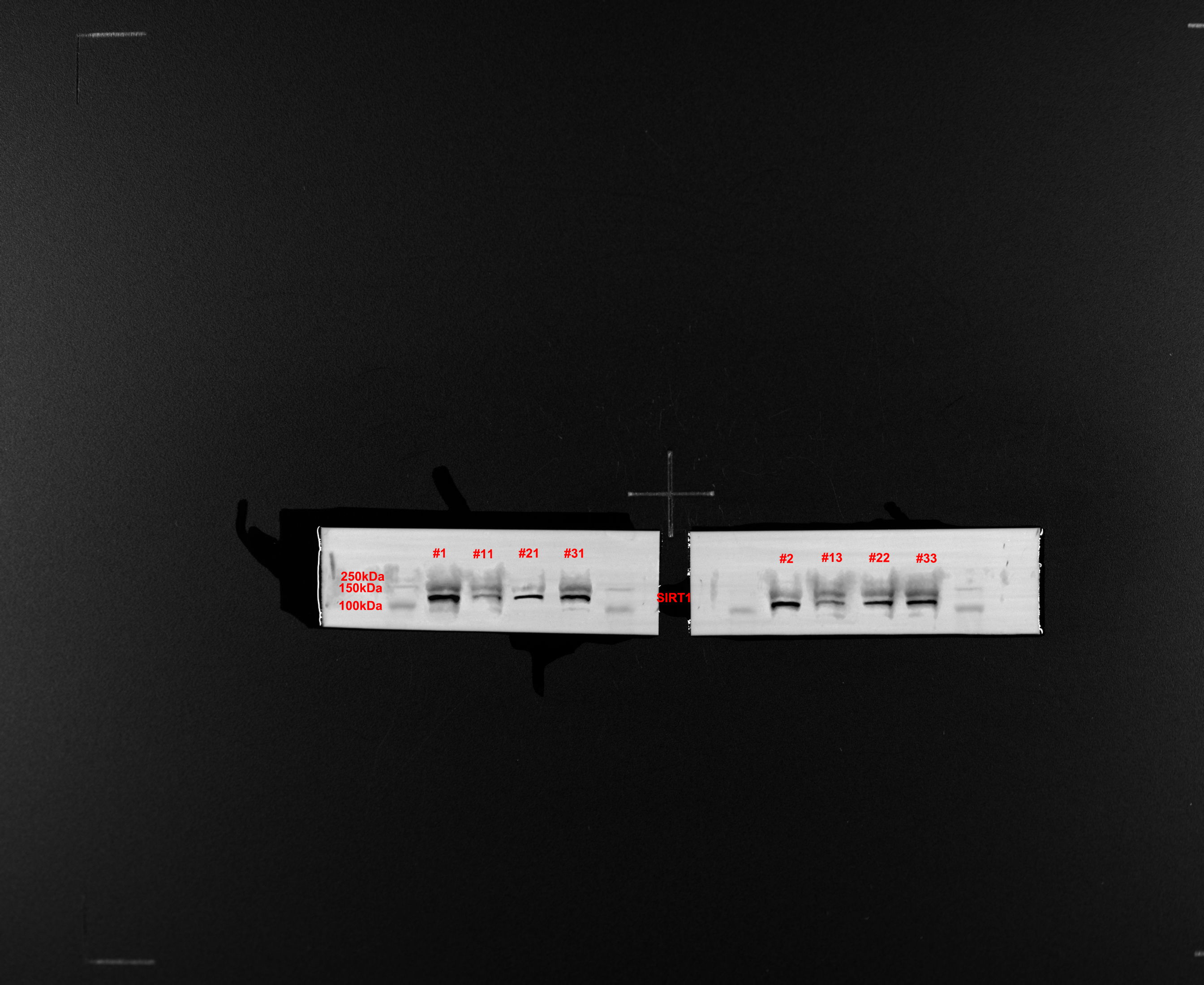

Supplement: Supplementary file 1 — Supplementary Material 1 [file 41065_2025_529_MOESM1_ESM.zip › raw data/SIRT1(1,11,21,31,2,13,22,33)+marker.jpg]

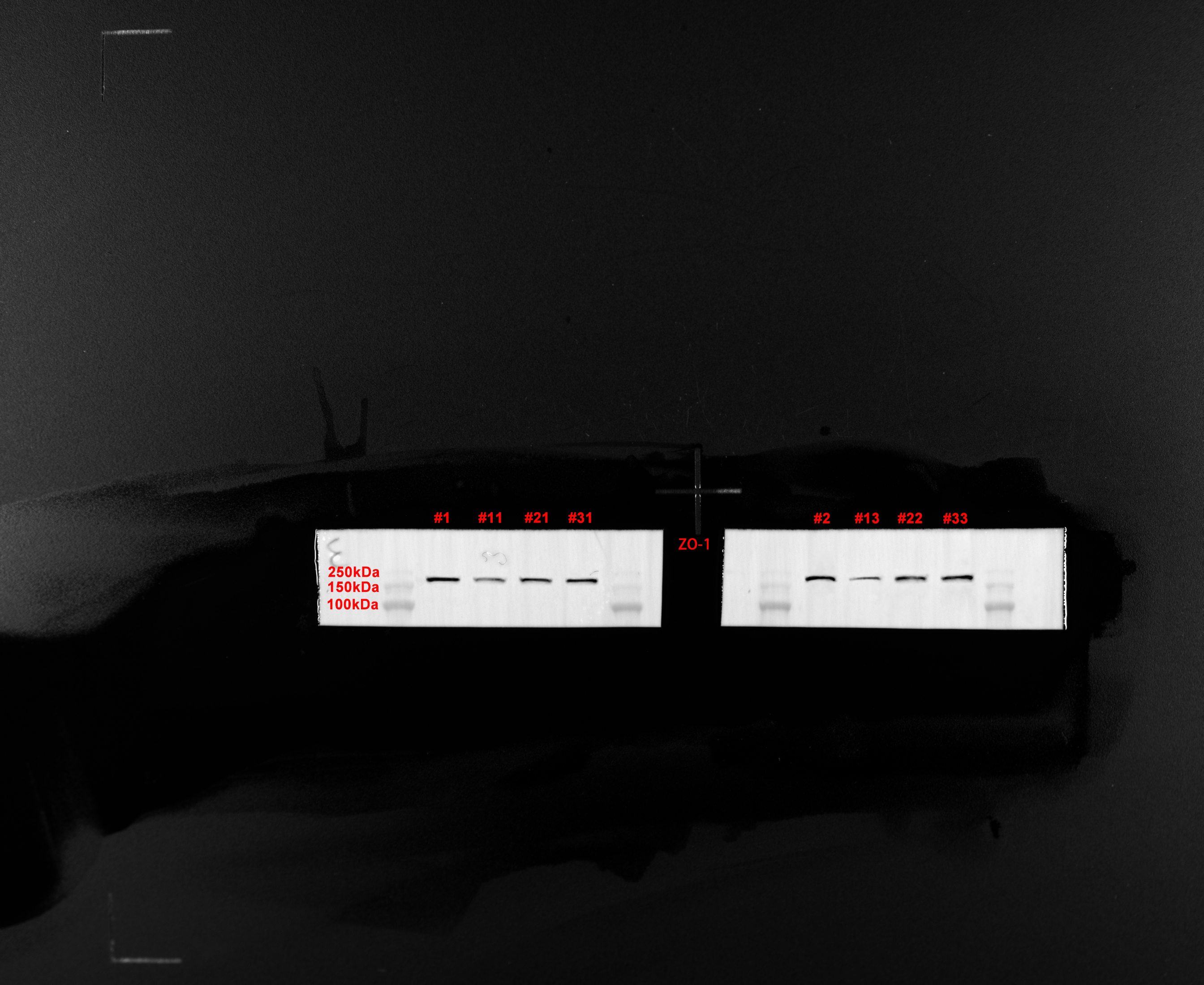

Supplement: Supplementary file 1 — Supplementary Material 1 [file 41065_2025_529_MOESM1_ESM.zip › raw data/ZO-1(1,11,21,31,2,13,22,33)+marker.jpg]
